# Генетическая характеристика клинических изолятов клебсиелл, циркулирующих в Новосибирске

**DOI:** 10.18699/VJ21.49-o

**Published:** 2021-03

**Authors:** A.V. Bardasheva, N.V. Fomenko, T.V. Kalymbetova, I.V. Babkin, S.O. Chretien, E.V. Zhirakovskaya, N.V. Tikunova, V.V. Morozova

**Affiliations:** Institute of Сhemical Biology аnd Fundamental Medicine of Siberian Branch of the Russian Academy of Sciences, Novosibirsk, Russia; JSC “Vector-Best”, Novosibirsk, Russia; JSC “Vector-Best”, Novosibirsk, Russia; Institute of Сhemical Biology аnd Fundamental Medicine of Siberian Branch of the Russian Academy of Sciences, Novosibirsk, Russia; Novosibirsk Research Institute of Traumatology and Orthopedics named after Ya.L. Tsivyan of the Ministry of Health of the Russian Federation, Novosibirsk, Russia; Institute of Сhemical Biology аnd Fundamental Medicine of Siberian Branch of the Russian Academy of Sciences, Novosibirsk, Russia; Institute of Сhemical Biology аnd Fundamental Medicine of Siberian Branch of the Russian Academy of Sciences, Novosibirsk, Russia; Institute of Сhemical Biology аnd Fundamental Medicine of Siberian Branch of the Russian Academy of Sciences, Novosibirsk, Russia

**Keywords:** Klebsiella, molecular serotyping, beta-lactam, fluoroquinolone, aminoglycoside, extended-spectrum beta-lactamases, metallo-beta-lactamases, disco-diffusion analysis, genetic determinants of antibiotic resistance, Klebsiella, молекулярное серотипирование, бета-лактамы, фторхинолоны, аминогликозиды, беталактамазы расширенного спектра, металло-бета-лактамазы, диско-диффузионный анализ, генетические детерминанты антибиотикорезистентности

## Abstract

Проанализированы 72 клинических штамма Klebsiella spp., изолированных в Новосибирске из образцов, полученных от людей. Проведена видовая идентификация штаммов по последовательностям генов 16S рРНК и rpoB. Показано, что в популяции клебсиелл доминировали штаммы Klebsiella pneumoniaе (57 штаммов), остальные 15 штаммов относились к видам K. grimontii, K. aerogenes, K. oxytoca и K. quasipneumoniae. Методом молекулярного серотипирования с использованием последовательности гена wzi штаммы K. pneumoniae были отнесены к двадцати одному K-cеротипу, при этом большую долю составляли вирулентные серотипы K1 и K2. Выявлено, что штаммы K. pneumoniae, полученные от госпитализированных пациентов, обладали максимально выраженной резистентностью к различным классам антибиотиков в отличие от остальных видов клебсиелл. Методом ПЦР в реальном времени обнаружено, что в исследованной популяции присутствуют гены семейств blaSHV, blaTEM, blaCTX и ген blaOXA-48 , являющиеся генетическими детерминантами резистентности к бета-лактамам. Показано, что присутствие последовательности blaCTX коррелирует с продукцией штаммом бета-лактамаз расширенного спектра, а фенотипическая устойчивость к карбапенемам обусловлена наличием гена blaOXA-48 . При этом генов карбапенемаз vim, ndm, kpc, imp обнаружено не было. Cреди исследованных генов устойчивости к аминогликозидам были найдены гены aph(6)-Id и aadA, однако их наличие не всегда совпадало с фенотипической резистентностью. Устойчивость к фторхинолонам у большинства штаммов сопровождалась присутствием генов aac(6’)-Ib-cr, oqxA, oqxB, qnrB и qnrS в различных комбинациях, при этом наличие только генов oqxA и/или oqxB не коррелировало с устойчивостью к фторхинолонам. Таким образом, обнаружение blaCTX и blaOXA-48 может быть использовано для быстрого выявления продукции беталактамаз расширенного спектра и определения резистентности клебсиелл к карбапенемам, а выявление генов aac(6’)-Ib-cr и/или qnrB/qnrS – для быстрого определения устойчивости к фторхинолонам

## Abstract (en)

72 clinical strains of Klebsiella spp. isolated from samples obtained from humans in Novosibirsk, Russia, were analyzed. Species identification of strains was performed using 16S rRNA and rpoB gene sequences. It was revealed that Klebsiella pneumoniae strains were dominant in the population (57 strains), while the remaining 15 strains were K. grimontii, K. aerogenes, K. oxytoca and K. quasipneumoniae. By molecular serotyping using the wzi gene sequence, K. pneumoniae strains were assigned to twenty-one K-serotypes with a high proportion of virulent K1- and K2-serotypes. It was found that K. pneumoniae strains isolated from the hospitalized patients had a higher resistance to antibiotics compared to the other Klebsiella species. Real-time PCR revealed that the population contained genes of the blaSHV, blaTEM, blaCTX families and the blaOXA-48 gene, which are the genetic determinants of beta-lactam resistance. It has been shown that the presence of the blaCTX sequence correlated with the production of extended-spectrum beta-lactamases, and phenotypic resistance to carbapenems is due to the presence of the blaOXA-48 gene. At the same time, the carbapenemase genes vim, ndm, kpc, imp were not detected. Among the aminoglycoside resistance genes studied, the aph(6)-Id and aadA genes were found, but their presence did not always coincide with phenotypic resistance. Resistance to fluoroquinolones in the vast majority of strains was accompanied by the presence of the aac(6’)-IB-cr, oqxA, oqxB, qnrB, and qnrS genes in various combinations, while the presence of the oqxA and/or oqxB genes alone did not correlate with resistance to fluoroquinolones. Thus, the detection of blaCTX and blaOXA-48 can be used to quickly predict the production of extended-spectrum beta-lactamases and to determine the resistance of Klebsiella to carbapenems. The detection of the aac(6’)-Ib-cr and/or qnrB/qnrS genes can be used to quickly determine resistance to fluoroquinolones.

## Введение

Клебсиеллы представляют собой род грамотрицательных инкапсулированных неподвижных бактерий, относящихся к семейству Enterobacteriaceae порядка Enterobacteriales. Род насчитывает более 12 видов, из которых чаще всего встречаются Klebsiella pneumoniae и K. oxytoca. Клебсиеллы являются частью нормальной микробиоты желудочно-кишечного тракта, кожных покровов и верхних дыхательных путей здоровых людей (Broberg et al., 2014). В то же время они служат одной из наиболее распространенных причин как нозокомиальных, так и внебольничных инфекций, включая инфекции мочевыводящих путей, бактериемию, пневмонию, неонатальный абсцесс и гнойный абсцесс печени (Podschun, Ullmann, 1998; Mukherjee et al., 2020).

Для лечения пациентов с клебсиельными инфекциями используют бета-лактамные антибиотики, фторхинолоны и аминогликозиды (Galal et al., 2019). При этом основными в терапии являются бета-лактамы, которые блокируют синтез клеточной стенки бактерий и наименее токсичны для человека. Применение данного класса антибиотиков ограничено способностью многих штаммов клебсиелл продуцировать бета-лактамазы, расщепляющие бета-лактамные антибиотики (Козлова и др., 2018). Традиционно существуют две классификации бета-лактамаз – функциональная и структурная. Первая основана на их способности расщеплять различные классы бета-лактамов и на чувствительности к ингибиторам, таким как клавулановая кислота, сульбактам и тазобактам (функциональные группы 1, 2 и 3). Вторая классификация (структурная) распределяет лактамазы по молекулярным классам A, B, C и D соответственно сходству и различиям их белковых последовательностей (Bush, Jacoby, 2010). Серьезную проблему с практической точки зрения представляют бета-лактамазы расширенного спектра действия (БЛРС), относящиеся ко второй функциональной группе. Они характеризуются способностью расщеплять различные классы бета-лактамов, включая пенициллины, цефалоспорины и карбапенемы. К БЛРС относятся ферменты семейств TEM, SHV, CTX-M, OXA и др. (Gharrah et al., 2017; Galal et al., 2019). На сегодняшний день для каждого семейства бета-лактамаз известно большое количество аллельных вариантов, обеспечивающих устойчивость к третьему поколению цефалоспоринов, монобактамам и карбапенемам (Liakopoulos et al., 2016). Помимо функциональной и структурной классификации, ферменты, гидролизующие бета-лактамы, разделяются на два типа по механизму действия: сериновые бета-лактамазы и металло-бета-лактамазы (МБЛ), требующие двухвалентных катионов, обычно цинка, в качестве кофакторов (Walsh et al., 2005). Известно около десяти семейств металло-бета-лактамаз, а именно: IMP, VIM, SPM, GIM, SIM, AIM, KHM, NDM, TMB и др. Наиболее широко распространены лактамазы семейств IMP, VIM и NDM (Тапальский и др., 2012).

Для лечения инфекций, вызванных грамотрицательными бактериями, наряду с бета-лактамными антибиотиками используют аминогликозиды, которые способны связывать молекулы антибиотика с субъединицей 16S рРНК бактерии и ингибировать синтез белка. Самый распространенный механизм бактериальной резистентности к аминогликозидам – продукция aминогликозид-модифицирующих ферментов. Аминогликозид-модифицирующие ферменты представляют собой фосфотрансферазы (APH), ацетилтрансферазы (AAC) или нуклеотидилтранферазы (ANT) (Ramirez, Tolmasky, 2010).

Еще одним используемым классом антибиотиков являются фторхинолоны, которые воздействуют на ДНКгиразу и ДНК-топоизомеразу IV (Машковский, 2005). К основным механизмам резистентности грамотрицательных бактерий к данным антибиотикам относятся модификация антибиотика, например с использованием плазмид-кодируемой аминогликозидацетилтрансферазы AAC (6′)-Ib-cr, защита мишени (семейство плазмидных генов qnr) и система эффлюкса, например OqxAB-TolC RND, гены которой находятся в хромосоме большинства штаммов K. pneumoniae (Yang et al., 2014; Hooper, Jacoby, 2015).

В последние годы заметной проблемой стал рост резистентности клебсиельных штаммов, в особенности внутрибольничных изолятов, ко всем клинически значимым антибиотикам. Именно резистентность делает клебсиелл лидерами среди оппортунистических патогенов (Чеботарь и др., 2020). Цель нашего исследования – анализ генетических и фенотипических маркеров резистентности, включая бета-лактамы, фторхинолоны и аминогликозиды, клинических изолятов клебсиелл, выделенных в г. Новосибирске (Россия).

## Материалы и методы

**Бактериальные штаммы.** В работе исследовали 72штам-ма бактерий рода Klebsiella, выделенных в Новосибирске из клинических образцов. Чистые культуры бактерий получали, как описано ранее (Козлова и др., 2017); принадлежность к роду Klebsiella определяли по культурально-морфологическим признакам с использованием селективных сред – агара МакКонки (BioMerieux, Франция) и агара Левина (OXOID, Великобритания). Штаммы депонировали в Коллекции экстремофильных микроорганизмов и типовых культур Института химической биологии и фундаментальной медицины (КЭМТК ИХБФМ) СО РАН (http://www.niboch.nsc.ru/doku.php/emtc_collection).

**Идентификация бактериальных штаммов.** Видовую принадлежность бактерий устанавливали секвенированием последовательностей гена 16S рРНК (1342 п. н.) (Козлова и др., 2017) и подтверждали секвенированием фрагмента гена rpoB (501 п.н.) (Morozova et al., 2019). Для определения вида использовали наиболее близкие референсные последовательности рода Klebsiella, присутствующие в базе данных NCBI GenBank (https://www.ncbi.nlm.nih.gov), при уровне сходства последовательностей не менее 98 %. Последовательности генов 16S рРНК депонировали в базу данных NCBI GenBank под номерами МТ436838–MT436841, MT436848, MT436849, MT436851– MT436856, MT439052–MT439054, MT439056–MT439058, MT439061–MT439079, MT439081–MT439103, MT439106, MT489341–MT489346. Последовательности генов rpoB депонировали в базу данных NCBI GenBank под номерами MT447755–MT447758, MT447760–447768, MT447770, MT447771, MT447773–MT447775, MT447778–MT447798, MT447825, MT447828–MT447830.

**Молекулярное серотипирование штаммов K. pneumoniae и K. quasipneumoniae.** Серотипирование коллекционных штаммов проводили путем секвенирования последовательностей гена wzi, относящегося к кластеру генов синтеза полисахаридной капсулы клебсиелл, как описано ранее (Brisse et al., 2013; Morozova et al., 2019). Для определения серотипа использовали наиболее близкие последовательности гена wzi референсных штаммов K. pneumoniae, присутствующие в базе данных NCBI GenBank. В ходе работы молекулярное серотипирование было проведено для 13 коллекционных штаммов K. pneumoniae и K. quasipneumoniae. Последовательности генов wzi были депонированы в базу данных NCBI GenBank под номерами MT434694, MT447742–MT4477754. Остальные коллекционные штаммы K. pneumoniae и K. quasipneumoniae были типированы ранее (Morozova et al., 2019).

**Оценку гипермукоидности штаммов** проводили, используя струнный тест (Lee H.C. et al., 2006). Для этого стандартной бактериологической петлей растягивали слизистый тяж из бактериальных колоний, выращенных на кровяном агаре с 5 % овечьей крови, при 37 °С в течение ночи. При формировании вязкой нити длиной >10 мм штаммы оценивались как гипермукоидные.

**Определение фенотипической антибиотикорезистентности штаммов.** Антибиотикорезистентность штаммов выявляли диско-диффузионным методом согласно рекомендациям Европейского комитета по определению чувствительности к антимикробным препаратам (EUCAST, https://eucast.org) с использованием агара Мюллера–Хинтона (OXOID, Великобритания). Исследовали чувствительность штаммов к следующим антибиотикам (OXOID): амоксициллин/клавулановая кислота (АMP/CAL, 20/10 мкг); ампициллин/сульбактам (AMP/ SUL, 10/10 мкг); пиперациллин/тазобактам (PIP/TZB, 30/10 мкг); цефтазидим (CAZ, 10 мкг); амикацин (AMK, 30 мкг); гентамицин (GEN, 10 мкг); левофлоксацин (LEV, 5 мкг); ципрофлоксацин (CIP, 5 мкг); имипенем (IPM, 10 мкг); меропенем (MEM, 10 мкг); хлорамфеникол (CLM, 30 мкг); азтреонам (ATM, 30 мкг). Штамм E. coli ATCC 25922, не обладающий резистентностью к антибиотикам, использовали в качестве контроля.

**Фенотипическое определение БЛРС.** Выявление БЛРС, резистентных к клавуланат-защищенным пенициллинам, проводили, как описано ранее (Hoa et al., 1998; Эйдельштейн, 2001). Штамм K. pneumoniae ATCC 700603, продуцирующий БЛРС, использовали в качестве положительного контроля

**Выявление продукции металло-бета-лактамаз.** Продукцию МБЛ штаммами клебсиелл исследовали методом двойных дисков с использованием этилендиаминтетраацетата натрия (ЭДТА) по методу, описанному ранее (Lee K. et al., 2001; Yong et al., 2002; Иванов, Егоров, 2008). Увеличение на 8 мм зоны ингибирования роста вокруг диска, содержащего антибиотик и ЭДТА, по сравнению с исходным диском с антибиотиком, интерпретировали как положительный результат (Yong et al., 2002).

**Выявление генов антибиотикорезистентности методом ПЦР в реальном времени.** Выделение нуклеиновых кислот проводили из 100 мкл суспензии клеток Klebsiella spp. с помощью набора реагентов «РеалБест ДНК-экспресс» (АО «Вектор-Бест», Россия) в соответствии с инструкцией производителя. Гены резистентности выявляли с использованием олигонуклеотидов, представленных в табл. 1. Также штаммы анализировали на наличие генов бета-лактамаз семейства ОXA: blaOXA-2, blaOXA-10, blaOXA-23, blaOXA-24, blaOXA-58; генов устойчивости к карбапенемам семейств kpc, vim, ndm, imp; генов устойчивости к аминогликозидам aac3, aph6, ant2, ant6; гена устойчивости к фторхинолонам qnrA. Перечисленные гены обнаружены не были, и последовательности соответствующих олигонуклеотидов не приводятся.

**Table 1.**
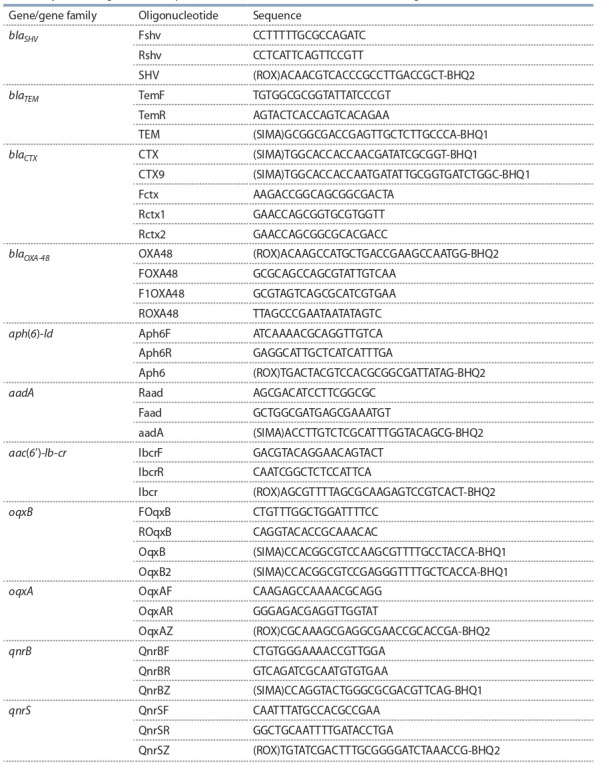
Synthetic oligonucleotide primers for the detection of antibiotic resistance genes

ПЦР в реальном времени осуществляли с использованием амплификатора с флуоресцентной детекцией CFX96 Touch™ Real-Time PCR Detection System (Bio-Rad, США). Условия амплификации: прогрев при 94 °С – 1 мин, далее 50 циклов (94 °С – 10 с, 60 °С – 20 с).

## Результаты

**Видовая принадлежность, молекулярное серотипирование и выявление гипермукоидности**

Из 1512 клинических образцов от пациентов с разными заболеваниями было выявлено 72 (4.8 %) образца, содержащих клебсиеллы. Из них 46 штаммов были выделены из образцов от амбулаторных больных, а 26 штаммов – от госпитализированных пациентов. В результате видовой идентификации с использованием генов 16S рРНК и rpoВ cреди штаммов от амбулаторных больных было выделено 34 штамма K. pneumoniae (74 %), 5 – K. grimontii, 4 – K. aerogenes, 2 – K. oxytoca и 1 – K. quasipneumoniae. Среди штаммов от госпитализированных пациентов было 23 штамма K. pneumoniae (88 %), 2 – K. grimontii и 1 – K. aerogenes.

Методом молекулярного серотипирования по последовательности гена wzi был выявлен 21 K-серотип (табл. 2). К наиболее вирулентным серотипам K1 и K2 принадлежали 15 штаммов от амбулаторных больных и 7 штаммов от госпитализированных пациентов. Из образцов, взятых от пациентов с диареей при поступлении в Инфекционную клиническую больницу № 1 г. Новосибирска, было выявлено 4 штамма K47-cеротипа. Еще 11 штаммов (серотипы K22/K30 и K17) были получены от пациентов клиники Новосибирского НИИ травматологии и ортопедии им. Я.Л. Цивьяна (ННИИТО). K-серотип некоторых штаммов определить не удалось; можно было лишь заключить, что данный штамм относится к некой группе серотипов (см. табл. 2). По-видимому, для таких штаммов необходимо проводить серотипирование иммунологическими методами или секвенировать весь кластер генов синтеза полисахаридной капсулы (35 тыс. п.н.).

**Table 2.**
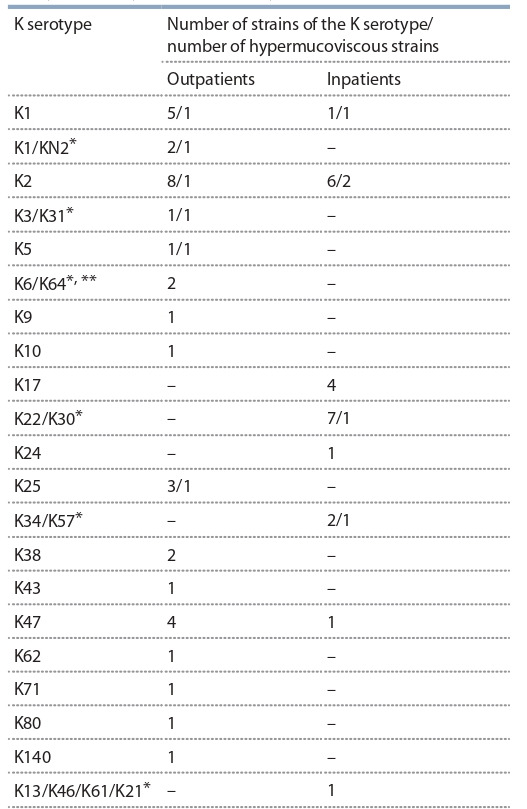
K. pneumoniae and K. quasipneumoniae strains: serotypes and hypermucoviscosity Notе. K serotypes were identified by molecular serotyping with the sequence of the wzi gene. * The wsi sequence is insufficient for precise serotyping. ** One of the strains (KQ_1250) belonged to K. quasipneumoniae.

Количество гипермукоидных среди штаммов K. pneumoniae от амбулаторных и госпитализированных больных составило 13 и 19 % соответственно. Этот фактор патогенности присутствовал у штаммов, относившихся к разным K-серотипам (см. табл. 2).

**Фенотипическая резистентность к антибиотикам**

Среди штаммов от амбулаторных больных чаще выявлялась резистентность к ампициллин/клавуланату (более 40 % случаев), почти не встречалась резистентность к карбапенемам, БЛРС продуцировали 28 % штаммов. Штаммов, продуцирующих МБЛ, не выявлено. Среди штаммов от госпитализированных пациентов резистентность к разным классам антибиотиков оказалась выше (см. рисунок). Свыше 70 % штаммов были нечувствительны к защищенным пенициллинам, более 40 % проявляли резистентность к меропенему, 11.5 и 60 % штаммов являлись продуцентами МБЛ и БЛРС соответственно. Также среди штаммов от амбулаторных и госпитализированных пациентов было выявлено соответственно 21.7 и 54 % полирезистентных штаммов, обладавших устойчивостью к четырем классам антибиотиков.

**Fig. 1.**
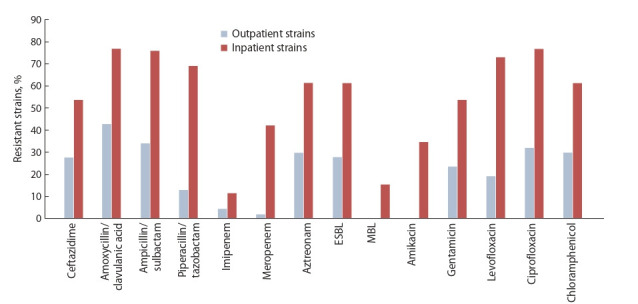
Phenotypic antibiotic resistance in clinical Klebsiella strains.

**Генетические детерминанты резистентности штаммов от амбулаторных больных**

**Штаммы, чувствительные к использованнымантибиотикам.** В ходе исследования было показано, что 30 (65 %) штаммов Klebsiella spp., полученных от амбулаторных больных, чувствительны к использованным антибиотикам либо резистентны только к одному-двум из них (табл. 3). В то же время у большинства штаммов были обнаружены гены бета-лактамаз семейства shv, а также хромосомные гены oqxA и oqxB системы эффлюкса, которая должна способствовать выведению фторхинолонов из клетки. Наличие генов oqxA и oqxB у чувствительных штаммов согласуется с литературными данными, так как известно, что эти гены присутствуют у большинства штаммов K. pneumoniae (Yang et al., 2014; Hooper, Jacoby, 2015). Штамм KP_2634 содержал ген aadA, но был чувствителен к аминогликозидам, тогда как резистентный к ципрофлоксацину штамм KP_3425 обладал, помимо генов oqxA и oqxB, плазмидным геном qnrS (см. табл. 3). По-видимому, присутствие одиночных генов резистентности в клетках клебсиелл не всегда коррелирует с про-явлением фенотипической резистентности, наличие которой зависит от экспрессии этих генов или может быть опосредовано другими механизмами.

**Table 3.**
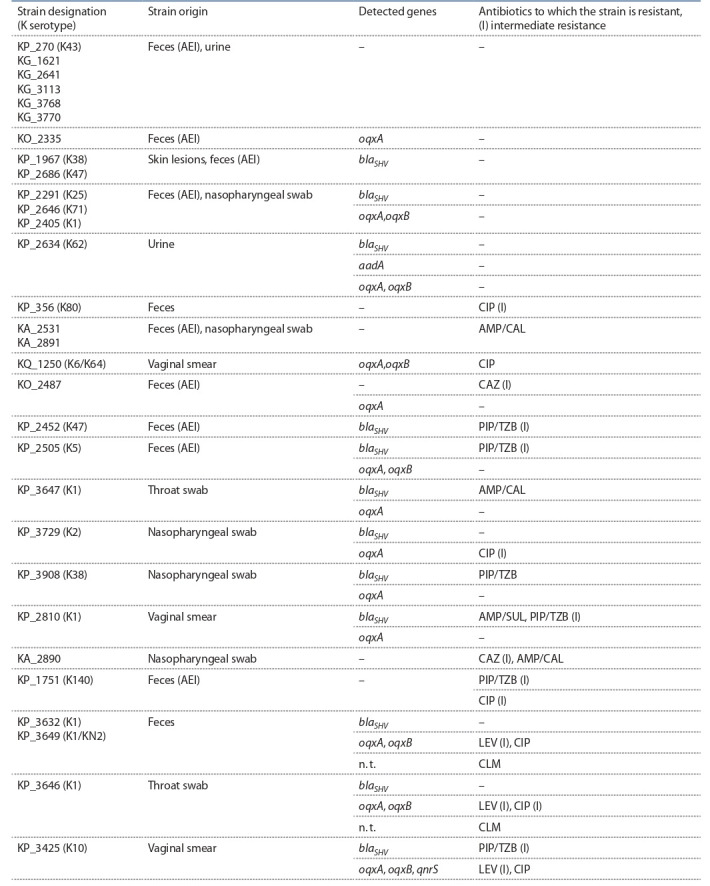
Resistance genes found in outpatient strains sensitive to antibiotics tested Note. Here and in Tables 4 and 5: KA – K. aerogenes, KG – K. grimontii, KO – K. oxytoca, KP – K. pneumoniae, KQ – K. quasipneumoniae; AEI, acute enteric infection; DFD, diabetic foot disease. Strain numbers correspond to numbers in the Collection of extremophilic microorganisms and type cultures, Institute of Chemical Biology and Fundamental Medicine, Novosibirsk. Strains were tested for sensitivity to antibiotics: amoxycillin+clavulanic acid (AMP/CAL, 20/20 μg), ampicillin/sulbactam (AMP/SUL, 10/10 μg), piperacillin/tazobactam (PIP/TZB, 30/10 μg), ceftazidime (CAZ, 10 μg), amikacin (AMK, 30 μg), gentamicin (GEN, 10 μg), levofloxacin (LEV, 5 μg), ciprofloxacin (CIP, 5 μg), imipenem (IPM, 10 μg), meropenem (MEM, 10 μg), chloramphenicol (CLM, 30 μg), aztreonam (ATM, 30 μg); n.t. — not tested.

**Штаммы, устойчивые к использованным антибиотикам.** В эту группу были объединены штаммы, устойчивые к трем и более антибиотикам. При этом устойчивость к двум или более защищенным пенициллинам (АMP/CAL, AMP/SUL, PIP/TZB) сопровождалась наличием генов семейств blaSHV и blaTEM у всех штаммов, кроме KA_2420, KP_2826 и KP_2827. Присутствие гена семейства blaCTX во всех случаях сопровождалось резистентностью к цефтазидиму (CAZ) и продукцией БЛРС. Резистентность к карбапенемам была выявлена в двух штаммах, из них KP_3597 содержал ген blaOXA-48 , а в штамме KA_2420 не обнаружено никаких генов резистентности из изучаемого спектра (табл. 4).

**Table 4.**
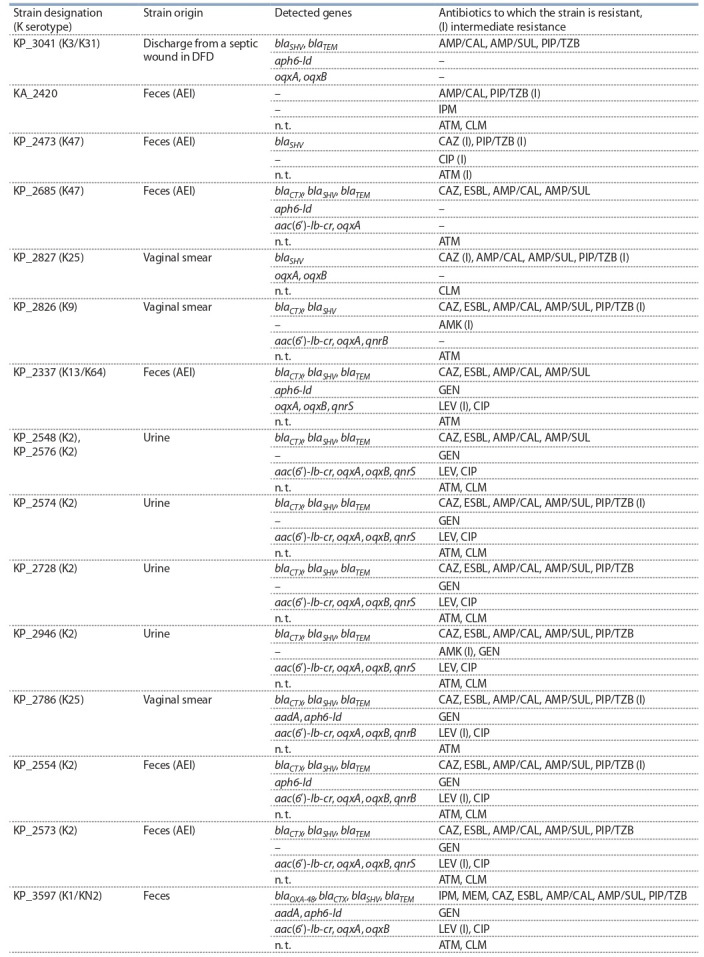
Resistance genes found in outpatient strains resistant to antibiotics tested

Резистентность к аминогликозидам (AMK и/или GEN) присутствовала у 11 штаммов, но только у четырех из них были найдены последовательности генов aadA и/или aph6-Id. Последовательность aph6-Id выявлялась и у штаммов, не обладавших резистентностью к аминогликозидам (см. табл. 4)

Полная (R) или промежуточная (I) резистентность к фторхинолонам (LEV и/или CIP) была выявлена у 11 штаммов; у 10 из них были найдены гены aac(6′)-Ib-cr, oqxA, oqxB, qnrB и qnrS в различных комбинациях. Надо отметить, что присутствие только генов oqxA и/или oqxB не коррелирует с наличием резистентности к фторхинолонам (см. табл. 3 и 4).

**Генетические детерминанты резистентности штаммов от госпитализированных пациентов**

Из 26 штаммов только пять не обладали множественной устойчивостью к антибиотикам, и 42 % штаммов были резистентны к карбапенемам (табл. 5). Как и в штаммах от амбулаторных больных, устойчивость к цефтазидиму и продукция БЛРС совпадали с присутствием гена семейства blaCTX, а устойчивость к карбапенемам была связана с наличием гена blaOXA-48. Ген blaOXA-48 также был ассоциирован с продукцией БЛРС у штаммов KP_3521, KP_3522, KP_3533, у которых blaCTX не выявлялся. Генов карбапенемаз семейств vim, ndm, kpc, imp не было обнаружено, хотя штаммы KP_3521, KP_3526 и KP_3533 обладали резистентностью к карбапенемам, подавляемой в присутствии ЭДТА (см. табл. 5). По-видимому, резистентность к карбапенемам обусловлена другими генетическими механизмами

**Table 5.**
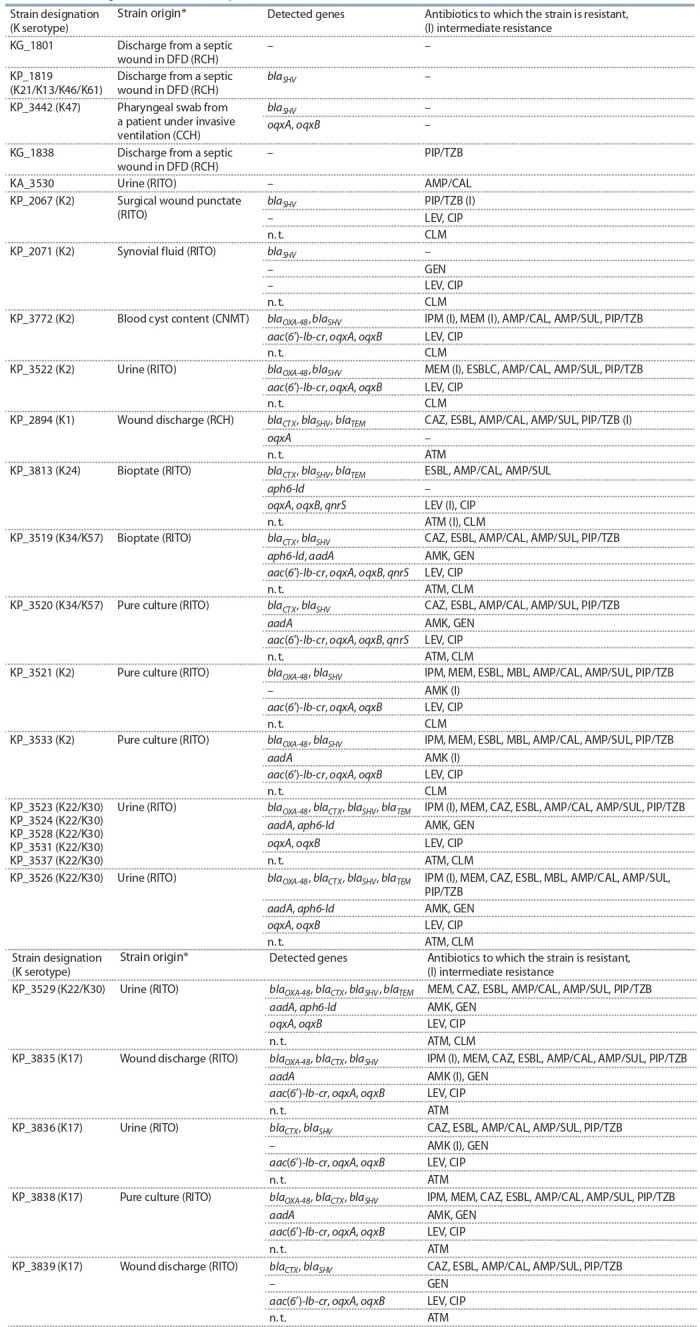
Resistance genes in strains from inpatients * RCH, Railway Clinical Hospital, Novosibirsk-Glavnyy Railway Station; CCH, Central Clinical Hospital, Siberian Branch of the RAS; RITO, Research Institute of Traumatology and Orthopedics, Novosibirsk; CNMT, Center for New Medicinal Technologies, Novosibirsk.

Шестнадцать штаммов от госпитализированных пациентов (61 %) были устойчивы к аминогликозидам (AMK и/или GEN), у 12 из них были выявлены гены aadA и/или aph6-Id. Устойчивость оставшихся 4 штаммов была обусловлена другими механизмами, так как у них не обнаружены исследуемые гены резистентности (см.табл. 5).

Резистентность к фторхинолонам (LEV и CIP) была выявлена у 20 штаммов, у 18 из них найдены гены aac(6′)-Ib-cr, oqxA, oqxB и qnrS в различных комбинациях. Так же, как и в популяции штаммов от амбулаторных больных, выявление только генов oqxA и/или oqxB не коррелировало с наличием резистентности к фторхинолонам (см. табл. 3–5).

## Обсуждение

В ходе исследования среди 72 клинических штаммов клебсиелл было выявлено 57 штаммов K. pneumoniae. Из 15 оставшихся штаммов семь были отнесены к K. grimontii, пять – к K. aerogenes, два – к K. oxytoca и один штамм – к K. quasipneumoniae (см. табл. 3–5). Четырнадцать из этих 15 штаммов показали чувствительность к большинству использованных антибиотиков. Исключением стал штамм K. aerogenes KA_2420, устойчивый к защищенным пенициллинам, имипенему, азтреонаму и хлорамфениколу (см. табл. 4). При этом ни у одного штамма, включая KA_2420, не выявлено исследуемых генов резистентности, кроме oqxA, oqxB у штаммов KO_2335, KO_2487 и KQ_1250. Возможно, генетические механизмы резистентности различны у штаммов клебсиелл, относящихся к разным видам. 

Основную проблему для терапии представляют штаммы K. pneumoniae, поскольку именно они являются полирезистентными (см. табл. 5). Анализ 57 штаммов K. pneumoniae на наличие генов бета-лактамаз семейств blaSHV, blaTEM, blaCTX и бета-лактамаз семейства OXA (blaOXA-2, blaOXA-10, blaOXA-23, blaOXA-24, blaOXA-48 и blaOXA-58) выявил гены первых трех семейств и ген blaOXA-48 . Ген blaSHV присутствовал у 84 % амбулаторных штаммов K. pneumoniae со слабовыраженной резистентностью (табл. 3, 6) и у 100 % резистентных штаммов как от амбулаторных, так и от госпитализированных больных (см. табл. 4–6). Ген blaTEM отсутствовал у чувствительных к антибиотикам штаммов от амбулаторных больных, но был обнаружен у 80 % резистентных штаммов от амбулаторных больных и лишь у 40 % штаммов от госпитализированных пациентов (см. табл. 4–6). Наличие blaSHV и/или blaTEM не всегда сочеталось с резистентностью к исследованным бета-лактамам, поэтому они не могут быть корректными маркерами резистентности, в отличие от blaCTX и blaOXA-48 (см. табл. 4 и 5), выявление которых может быть использовано для быстрого определения продукции БЛРС и резистентности к карбапенемам соответственно.

**Table 6.**
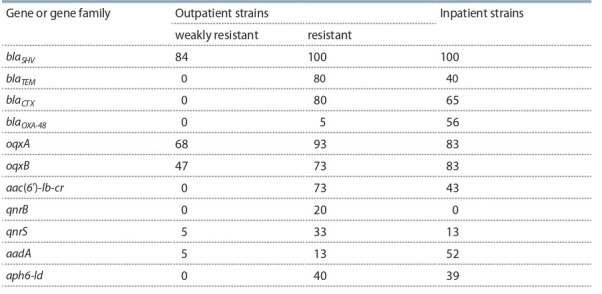
Percentage of K. pneumoniae strains bearing resistance genes

Согласно многоцентровому эпидемиологическому исследованию, проведенному в России в 2015–2016 гг., 75.6 % нозокомиальных штаммов K. pneumoniae продуцировали БЛРС, 26.5 % штаммов продуцировали карбапенемазы (OXA-48 – 21.5 %, NDM – 4.3, OXA-48 и NDM – 0.6, KPC – 0.1 %) (Сухорукова и др., 2019). В изученной нами выборке штаммов от госпитализированных пациентов доля штаммов-продуцентов БЛРС была похожей (65 %), но гораздо выше оказалась доля штаммов, устойчивых к карбапенемам и содержащих blaOXA-48 (56 %); при этом генов устойчивости к карбапенемам семейств vim, ndm, kpc, imp нами не выявлено.

Cреди исследованных генов устойчивости к аминогликозидам aac3, aph6, ant2, ant6, aph(6 )-Id, aadA были обнаружены только последние два. Их присутствие не всегда коррелировало с фенотипической резистентностью (см. табл. 3–5), свидетельствуя о том, что резистентность к аминогликозидам может быть обусловлена и другими механизмами

Из 22 штаммов K. pneumoniae, обладавших генами aac(6′ )-Ib-cr и/или qnrB/qnrS, резистентность к фторхинолонам (LEV и CIP) была определена у 20 (90 %) штаммов (см. табл. 3–5). Следует отметить, что ген qnrB обнаружен лишь в трех случаях (см. табл. 4 и 6) и, возможно, является малораспространенным в новосибирских штаммах. Тем не менее выявление aac(6′ )-Ib-cr и/или qnrB/qnrS хорошо согласуется с наличием фенотипической резистентности и может быть использовано для быстрого предсказания устойчивости изолята к фторхинолонам.

Считается, что плазмидные гены резистентности к фторхинолонам семейства qnr часто ассоциированы с БЛРС-продуцирующими изолятами (Robicsek et al., 2006). В нашем исследовании такая ассоциация была выявлена среди штаммов, выделенных от амбулаторных больных, так как 10 из 12 штаммов-продуцентов БЛРС содержали гены qnrB и/или qnrS (см. табл. 4). В то же время только 3 из 18 штаммов-продуцентов БЛРС, полученных от госпитализированных пациентов, содержали ген qnrS, при этом 17 из них были резистентны к фторхинолонам. Это свидетельствует о других возможных механизмах резистентности, таких как уменьшение проницаемости мембраны и сверхактивность эффлюксной помпы.

## Заключение

Таким образом, среди клинических штаммов клебсиелл, выделенных у пациентов в г. Новосибирске, доминировали штаммы K. pneumoniae. Обнаружены также штаммы K. grimontii, K. aerogenes, K. oxytoca и K. quasipneumoniae. Методом молекулярного серотипирования штаммы K. pneumoniae были отнесены к 21 K-cеротипу; большинство среди них составляли вирулентные серотипы K1 и K2. Выявлено, что штаммы K. pneumoniae обладали наибольшей резистентностью к антибиотикам среди различных видов клебсиелл. Генетическими детерминантами резистентности к бета-лактамам в исследованной популяции являлись blaSHV, blaTEM, blaCTX и blaOXA-48. Показаны ассоциации между присутствием blaCTX, blaOXA-48 и продукцией БЛРС и устойчивостью к карбапенемам соот-ветственно. Cреди исследованных генов устойчивости к аминогликозидам были обнаружены гены aph(6)-Id и aadA, однако их наличие не всегда совпадало с фенотипической резистентностью. Резистентность к фторхинолонам у большинства штаммов сопровождалась присутствием генов aac(6′ )-Ib-cr, oqxA, oqxB, qnrB и qnrS в различных комбинациях. Следует отметить, что присутствие одних только генов oqxA и/или oqxB не коррелировало с наличием устойчивости к фторхинолонам.

## Conflict of interest

The authors declare no conflict of interest.
